# Ethnicity, household composition and COVID-19 mortality: a national linked data study

**DOI:** 10.1177/0141076821999973

**Published:** 2021-03-24

**Authors:** Vahé Nafilyan, Nazrul Islam, Daniel Ayoubkhani, Clare Gilles, Srinivasa Vittal Katikireddi, Rohini Mathur, Annabel Summerfield, Karen Tingay, Miqdad Asaria, Ann John, Peter Goldblatt, Amitava Banerjee, Myer Glickman, Kamlesh Khunti

**Affiliations:** 1 Office for National Statistics, Newport, UK; 2 Department of Public Health, Environments and Society, London School of Hygiene and Tropical Medicine, London, UK; 3 Nuffield Department of Population Health, Big Data Institute, University of Oxford, Oxford, UK; 4 Diabetes Research Centre, University of Leicester, Leicester, UK; 5 MRC/CSO Social and Public Health Sciences Unit, University of Glasgow, Glasgow, UK; 6 6Department of Non-communicable Disease Epidemiology, London School of Hygiene and Tropical Medicine, London, UK; 7 LSE Health, London School of Economics and Political Sciences, London, UK; 8 Swansea University, Swansea, UK; 9 UCL Institute of Health Equity, University College London, London, UK; 10 Institute of Health Informatics, University College London, London, UK

**Keywords:** Clinical, ethnic studies, housing and health, infectious diseases, public health

## Abstract

**Objective:**

To estimate the proportion of ethnic inequalities explained by living in a multi-generational household.

**Design:**

Causal mediation analysis.

**Setting:**

Retrospective data from the 2011 Census linked to Hospital Episode Statistics (2017-2019) and death registration data (up to 30 November 2020).

**Participants:**

Adults aged 65 years or over living in private households in England from 2 March 2020 until 30 November 2020 (n=10,078,568).

**Main outcome measures:**

Hazard ratios were estimated for COVID-19 death for people living in a multi-generational household compared with people living with another older adult, adjusting for geographic factors, socioeconomic characteristics and pre-pandemic health.

**Results:**

Living in a multi-generational household was associated with an increased risk of COVID-19 death. After adjusting for confounding factors, the hazard ratios for living in a multi-generational household with dependent children were 1.17 (95% confidence interval [CI] 1.06–1.30) and 1.21 (95% CI 1.06–1.38) for elderly men and women. The hazard ratios for living in a multi-generational household without dependent children were 1.07 (95% CI 1.01–1.13) for elderly men and 1.17 (95% CI 1.07–1.25) for elderly women. Living in a multi-generational household explained about 11% of the elevated risk of COVID-19 death among elderly women from South Asian background, but very little for South Asian men or people in other ethnic minority groups.

**Conclusion:**

Elderly adults living with younger people are at increased risk of COVID-19 mortality, and this is a contributing factor to the excess risk experienced by older South Asian women compared to White women. Relevant public health interventions should be directed at communities where such multi-generational households are highly prevalent.

## Research in context

### Evidence before this study

A systematic review by Sze and colleagues demonstrated that people of ethnic minority background in the UK and the USA have been disproportionately affected by the Coronavirus disease 2019 (COVID-19) compared to White populations. We reviewed all papers included within the above systematic review to identify studies which empirically explored potential mediating pathways underpinning ethnic inequalities in COVID-19. In addition, we searched PubMed for studies related to the association between household composition and COVID-19 risk, using the terms ‘household’, ‘COVID-19’ and ‘mortality’, ‘death’ or ‘infection’ on 1 December 2020. Previous research has demonstrated that household size is associated with COVID-19 risk, but there is a lack of studies based on nationally representative individual records that examine the links between household composition and COVID-19 risk. In addition, no study has focused on multigenerational households. While several studies have examined whether socio-demographic and economic factors are driving ethnic inequalities in COVID-19, no study has sought to explicitly quantify the contribution of household composition to the elevated risk of COVID-19 mortality in ethnic minority groups.

### Added value of this study

Using retrospective data from the 2011 Census linked to Hospital Episode Statistics and death registration data for England, we examined the relationship between household composition and COVID-19 mortality risk among elderly adults (≥65 years). Living in a multi-generational household was associated with an increased risk of COVID-19 death. The adjusted hazard ratios for living in a multi-generational household with dependent children were 1.17 (95% confidence interval [CI] 1.06–1.30) and 1.21 (95% CI 1.06–1.38) for elderly men and women. The hazard ratios for living in a multi-generational household without dependent children were 1.07 (1.01–1.13) for elderly men and 1.17 (95% CI 1.07–1.25) for elderly women. Using a causal mediation approach, we estimated whether the higher propensity to live in multi-generational households among ethnic minority groups contributed to their raised risk of COVID-19 death. We found that living in a multi-generational household explained around 11% of the elevated risk of COVID-19 death among elderly women from a South Asian background, but little for South Asian men or people in other ethnic minority groups.

### Implications of all the available evidence

Living in a multi-generational household is associated with an increased risk of COVID-19 infection and death. The increase in risk appears greater for elderly women than men living in a multi-generational household, and this is particularly the case when living with dependent children. It explains some of the excess COVID-19 mortality risk for women of South Asian background, but very little for men of South Asian background or people from other ethnic groups. Differences in household composition are therefore unlikely to be the main explanation of ethnic inequalities in COVID-19 outcomes, but may make an important contribution for some specific population subgroups, and may therefore be taken into account when prioritising vaccination. Relevant public health interventions (such as the provision of free accommodation to assist with self-isolation) should be considered to mitigate risks of infection spread within a household. Ensuring such interventions are accessible to communities where multi-generational households are highly prevalent (such as South Asian women) may be warranted.

## Introduction

People of ethnic minority background in the UK and the USA have been disproportionately affected by COVID-19^1–5^ compared to the White population, particularly Black and South Asian groups. While several studies have investigated whether adjusting for socio demographic and economic factors and medical history reduces the estimated difference in risk of mortality and hospitalisation,^6–8^ the reasons for the differences in the risk of experiencing harms from COVID-19 are still being explored.

One important driver of these ethnic inequalities may be differences in household structure between ethnic groups. Household composition varies substantially between ethnic groups, with some ethnic minority populations more likely to live in large, multi-generational households.^9^ While living in multi-generational households is associated with increased social capital,^10^ which could have beneficial health effects,^11^ it may also increase the risk of potential viral transmission.^12,13^ For older people, who are at greater risk of experiencing severe complications if infected, residing with younger people may represent an increase in exposure to infection, which could lead to an increased risk of hospitalisation and mortality from COVID-19. To the best of our knowledge, no study has yet examined whether the difference in household composition partly explains the elevated risk of COVID-19 mortality in ethnic minority groups.

In this study, we examined the relationship between household composition and COVID-19 mortality risk among elderly adults (≥65 years) in England, with a focus on multi-generational households (elderly adults living with younger adults or dependent children). We then investigated how the propensity to live in a multi-generation household varies across ethnic groups, and whether this heterogeneity contributes to the raised risk of COVID-19 mortality among ethnic minority groups compared to the White population.

## Methods

### Data

This retrospective cohort study was based on the 2011 Census of England linked to mortality registration data and Hospital Episodes Statistics (2017–2019). The study population included all usual residents of England aged 65 years or over in 2020, who had been enumerated in private households at the time of the 2011 Census (27 March 2011), had not moved to a care home by 2019 (identified by linking to the NHS Patient Register) and were still alive on 2 March 2020. We further excluded individuals who entered the UK in the year before the Census due to their higher propensity to leave the UK prior to the study period, and those aged over 100 years at the time of the Census. Our study population consisted of 10,078,568 individuals aged 65 years or over at 2 March 2020 (see Table 3 in Appendix 1 for details on the number of participants at each stage of the sample selection).

**Table 1.** Distribution of study variables, stratified by household composition.

		All		Single	Two-adult households	MGH without young children	MGH with young children	3 + older adults
		Mean	SD	Mean	Mean	Mean	Mean	Mean
Mortality	COVID-19 death	0.0039	0.0624	0.0030	0.0055	0.0033	0.0045	0.0032
	Other death	0.0225	0.1484	0.0182	0.0323	0.0161	0.0146	0.0204
Household composition	Two older adults	0.5502	0.4975					
	Single	0.3260	0.4688					
	MGH without young children	0.0975	0.2967					
	MGH with young children	0.0197	0.1388					
	Other 3 + adults	0.0065	0.0806					
Age	Age in 2020	75.2	7.6	74.2	78.0	72.6	72.6	76.0
Sex	Female	0.5396	0.4984	0.4822	0.6561	0.4866	0.4889	0.5040
	Population density	3536.2	3818.7	3118.1	3915.0	4190.1	5449.0	4320.3
Ethnicity	Bangladeshi	0.0023	0.0483	0.0011	0.0011	0.0065	0.0381	0.0016
	Black African	0.0045	0.0670	0.0027	0.0046	0.0092	0.0276	0.0112
	Black Caribbean	0.0086	0.0926	0.0060	0.0114	0.0124	0.0181	0.0128
	Chinese	0.0035	0.0593	0.0030	0.0026	0.0085	0.0088	0.0059
	Indian	0.0181	0.1334	0.0121	0.0088	0.0640	0.1064	0.0387
	Mixed	0.0044	0.0665	0.0035	0.0055	0.0051	0.0074	0.0121
	Other	0.0119	0.1085	0.0089	0.0084	0.0297	0.0625	0.0261
	Pakistani	0.0077	0.0876	0.0039	0.0031	0.0251	0.1071	0.0070
	White	0.9388	0.2398	0.9588	0.9546	0.8396	0.6241	0.8847
Region	East	0.1196	0.3245	0.1246	0.1155	0.1112	0.0929	0.1031
	East Midlands	0.0905	0.2869	0.0948	0.0878	0.0799	0.0725	0.0759
	London	0.1026	0.3035	0.0817	0.1094	0.1695	0.2243	0.1617
	North East	0.0519	0.2219	0.0523	0.0539	0.0465	0.0392	0.0436
	North West	0.1336	0.3402	0.1303	0.1400	0.1333	0.1239	0.1282
	South East	0.1726	0.3779	0.1776	0.1685	0.1646	0.1406	0.1729
	South West	0.1222	0.3275	0.1298	0.1194	0.0967	0.0844	0.1247
	West Midlands	0.1070	0.3091	0.1062	0.1047	0.1139	0.1328	0.1032
	Yorkshire and the Humbers	0.1000	0.3000	0.1028	0.1008	0.0845	0.0894	0.0866
Household deprivation	Not deprived	0.3525	0.4777	0.4198	0.2356	0.3832	0.3068	0.1958
	Deprived along 1 dimension	0.3840	0.4863	0.3712	0.4168	0.3525	0.3547	0.3738
	Deprived along 2 dimensions	0.2158	0.4114	0.1727	0.2911	0.1996	0.2302	0.2883
	Deprived along 3 dimensions	0.0447	0.2066	0.0346	0.0526	0.0597	0.0944	0.1246
	Deprived along 4 dimensions	0.0031	0.0556	0.0018	0.0038	0.0050	0.0139	0.0174
Urban/rural classification	Rural hamlets and isolated dwellings	0.0434	0.2038	0.0509	0.0317	0.0405	0.0395	0.0510
	Rural hamlets and isolated dwellings in a sparse setting	0.0042	0.0646	0.0048	0.0032	0.0036	0.0041	0.0057
	Rural town and fringe	0.1055	0.3072	0.1143	0.1006	0.0835	0.0595	0.0792
	Rural town and fringeï¿½in a sparse setting	0.0050	0.0708	0.0052	0.0056	0.0029	0.0033	0.0035
	Rural village	0.0740	0.2618	0.0852	0.0617	0.0583	0.0481	0.0652
	Rural villageï¿½in a sparse setting	0.0052	0.0716	0.0057	0.0047	0.0035	0.0031	0.0100
	Urban city and town	0.4362	0.4959	0.4406	0.4473	0.3945	0.3440	0.4106
	Urban city and town in a sparse setting	0.0022	0.0465	0.0021	0.0024	0.0014	0.0016	0.0031
	Urban major conurbation	0.2905	0.4540	0.2578	0.3061	0.3828	0.4688	0.3459
	Urban minor conurbation	0.0338	0.1806	0.0332	0.0367	0.0288	0.0280	0.0259
Approximated social grade	AB	0.1972	0.3979	0.2267	0.1480	0.1951	0.2139	0.1519
	C1	0.2961	0.4565	0.2920	0.3135	0.2667	0.2757	0.2774
	C2	0.2006	0.4004	0.2207	0.1487	0.2536	0.2242	0.2323
	D	0.2675	0.4427	0.2405	0.3156	0.2618	0.2525	0.2798
	E	0.0386	0.1926	0.0201	0.0743	0.0227	0.0337	0.0586
Health	Very good	0.2209	0.4148	0.2460	0.1790	0.2242	0.1990	0.2071
	Good	0.4464	0.4971	0.4655	0.4168	0.4505	0.3951	0.4126
	Fair	0.2525	0.4344	0.2221	0.3044	0.2445	0.2740	0.2679
	Poor	0.0661	0.2484	0.0546	0.0825	0.0665	0.1069	0.0843
	Very poor	0.0141	0.1180	0.0117	0.0172	0.0143	0.0251	0.0281
Long-term health problem or disability	Daily activities no limited	0.1234	0.3288	0.1002	0.1609	0.1150	0.1650	0.1956
	Daily activities limited a little	0.1937	0.3952	0.1717	0.2374	0.1714	0.1963	0.1853
	Daily activities limited a lot	0.6830	0.4653	0.7280	0.6017	0.7137	0.6387	0.6191
Hospital-based co-morbidities	Cancer	0.1009	0.3012	0.1059	0.0980	0.0896	0.0758	0.0738
	Cardiovascular disease	0.2829	0.4504	0.2703	0.3122	0.2612	0.2830	0.2029
	Digestive disorder	0.0193	0.1375	0.0190	0.0197	0.0197	0.0193	0.0165
	Mental health condition	0.0210	0.1435	0.0159	0.0319	0.0150	0.0153	0.0178
	Metabolic disorder	0.0854	0.2794	0.0784	0.0904	0.0983	0.1364	0.0738
	Musculoskeletal disorder	0.0989	0.2985	0.0883	0.1237	0.0810	0.0864	0.0598
	Neurological disorder	0.0178	0.1322	0.0173	0.0193	0.0163	0.0140	0.0145
	Renal disorder	0.0674	0.2508	0.0575	0.0885	0.0551	0.0614	0.0513
	Respiratory disorder	0.1375	0.3443	0.1259	0.1616	0.1241	0.1394	0.1049
Overcrowded		0.0313	0.1741	0.0141	0.0372	0.0630	0.2104	0.1682
IMD decile	1	0.0670	0.2501	0.0495	0.0902	0.0729	0.1343	0.0981
	2	0.0723	0.2590	0.0590	0.0883	0.0823	0.1235	0.0927
	3	0.0813	0.2733	0.0705	0.0950	0.0899	0.1102	0.0964
	4	0.0911	0.2877	0.0842	0.0997	0.0975	0.1037	0.1053
	5	0.1021	0.3028	0.0999	0.1050	0.1050	0.1013	0.1038
	6	0.1106	0.3136	0.1138	0.1074	0.1058	0.0959	0.1150
	7	0.1170	0.3214	0.1243	0.1088	0.1094	0.0866	0.1155
	8	0.1188	0.3235	0.1288	0.1064	0.1112	0.0867	0.1008
	9	0.1205	0.3256	0.1335	0.1035	0.1140	0.0807	0.0951
	10	0.1193	0.3242	0.1366	0.0958	0.1120	0.0770	0.0773
Level of highest qualification	No qualification	0.3740	0.4839	0.3271	0.4570	0.3519	0.4099	0.3994
	Level 1: 1–4 GCSE/O-Level	0.0926	0.2898	0.0968	0.0840	0.0985	0.0854	0.0911
	Level 2: 5 + GCSE/O levels	0.1048	0.3063	0.1092	0.0992	0.1050	0.0790	0.0931
	Apprenticeship	0.0587	0.2351	0.0686	0.0424	0.0623	0.0381	0.0448
	Level 3: 2 + A Levels or equivalent	0.0663	0.2489	0.0731	0.0534	0.0713	0.0583	0.0874
	Level 4 + : degree or above	0.2381	0.4259	0.2608	0.2070	0.2243	0.1983	0.2064
	Other	0.0655	0.2474	0.0643	0.0569	0.0866	0.1310	0.0776
Tenure of household	Owned outright	0.6197	0.4855	0.6621	0.5912	0.5398	0.3326	0.5195
	Owned with a mortgage	0.1952	0.3964	0.2109	0.1169	0.3196	0.4272	0.2303
	Shared ownership	0.0041	0.0641	0.0033	0.0057	0.0032	0.0049	0.0056
	Social rented from council	0.0659	0.2481	0.0437	0.1068	0.0512	0.0782	0.0828
	Other social rented	0.0550	0.2279	0.0352	0.0933	0.0375	0.0564	0.0634
	Private rented	0.0492	0.2162	0.0373	0.0682	0.0424	0.0912	0.0756
	Living rent-free	0.0109	0.1040	0.0075	0.0179	0.0063	0.0094	0.0229

MGH: multi-generational household; IMD: Index of Multiple Deprivation; GCSE: General Certificate of Secondary Education.

**Table 2.** Hazard ratios for COVID-19-related death for elderly adults (aged ≥65 years) in England, compared to living in a household with one other older adult, stratified by sex.

	Men		Women	
	Age-adjusted	Fully adjusted	Age-adjusted	Fully adjusted
Single	1.37	1.132	1.291	1.105
	[1.328–1.414]	[1.091–1.174]	[1.244–1.341]	[1.059–1.152]
Multi-generational household without children	1.304	1.065	1.322	1.156
	[1.242–1.369]	[1.009–1.125]	[1.234–1.417]	[1.073–1.246]
Multi-generational household with children	1.772	1.174	1.858	1.21
	[1.619–1.940]	[1.062–1.299]	[1.656–2.084]	[1.060–1.380]
3+ elderly adults	0.894	0.995	0.722	0.845
	[0.745–1.073]	[0.824–1.080]	[0.570–0.916]	[0.658–1.086]
Observations	405,182		443,650	
Concordance	0.740	0.826	0.755	0.852

Note: Hazard ratios compared to living in a household with one other older adult. Fully adjusted Cox regression models include geographical factors (region, population density, urban/rural classification), ethnicity, socioeconomic characteristics (IMD decile, household deprivation, educational attainment, social grade, household tenancy), health (self-reported health and disability from the Census, pre-existing conditions based on hospital contacts, number of hospital admissions, total days spent in hospital), a measure for overcrowding and property type.

**Table 3.** Sample selection and number of participants.

Sample	Number of people
All	53,483,456
Excluding non-usual residents	52,637,675
Excluding those who died before 2 March 2020	48,468,645
Excluding those who lived in Wales in 2019	45,842,599
Excluding those living in care home in 2019	45,306,953
Excluding those over 110 years old	45,306,378
Excluding those less than 65	10,078,568

To adjust for out-migration, we applied weights reflecting the probability of having remained in the country until March 2020 after being enumerated in March 2011, based on data from the NHS Patient Register and the International Passenger Survey (IPS). Further information on the data has already been published.^6^ All the variables used in the analysis, including their definitions and sources, are detailed in Table 4 in Appendix 1.

**Table 4.** Variables used for the statistical modelling.

Variable	Coding	Source
**Demographic factors**		
Single year of age	Second-order polynomial	2011 Census
Ethnicity	Black Caribbean, Black African, Bangladeshi, Pakistani, Indian, Chinese, White, Mixed, Other	2011 Census
Household composition	Single; Two elderly adults; Multi-generational household without dependent children; Multi-generational household with dependent children; three or more elderly adults	2011 Census
**Geographical variables**		
Government office region	Dummy variables representing local authority districts	NHS Patient register 2019
Urban-rural classification	Urban major conurbation, Urban minor conurbation, Urban city and town, Urban city & town in a sparse, Setting, Rural town and fringe, Rural village, Rural hamlet & isolated dwellings, Rural town & fringe in a sparse, setting, Rural village in a sparse setting Rural hamlet & isolated dwellings, in a sparse setting	2011 Census
Population density of Lower Super Output Area (LSOA)	Second-order polynomial, allowing for a different slope beyond the 99th percentile of the distribution to account for extreme values	
**Socioeconomic variables**		
Index of Multiple Deprivation (IMD), Welsh Index of Multiple Deprivation (WIMD) [28, 29]	Dummy variables representing deciles of deprivation	Department for Communities and Local Government/ Welsh Government
Household deprivation^a^	Not deprived, deprived in one dimension, deprived in two dimensions, deprived in three dimensions, deprived in four dimensions	2011 Census
Household tenure	Own outright, own with mortgage, social rented, private rented, other	2011 Census
Approximated social grade	Higher & intermediate managerial, administrative, professional occupations; Supervisory, clerical & junior managerial, administrative, professional occupations; Skilled manual occupations; Semi-skilled & unskilled manual occupations; Unemployed and lowest grade occupations	2011 Census
Level of highest qualification	Degree, A-level or equivalent, GCSE or equivalent, no qualification	2011 Census
Overcrowding	Overcrowded household (occupancy rating < −1)	2011 Census
Type of accommodation	Detached house, semi-detached house, terraced house, flat, other	2011 Census
**Occupational exposure variables**		
Key worker in the household	Yes, no	2011 Census
Household exposure to disease	Maximum ‘exposure to disease’ score within each household, ranging from 0 (no exposure) to 100 (maximum exposure), derived from O*NET data	2011 Census
Household proximity to others	Maximum of ‘proximity to others’ score within each household, ranging from 0 (no exposure) to 100 (maximum exposure), derived from O*NET data	2011 Census
**Health-related variables**		
Self-reported health status	Very good, good, fair, poor, very poor	2011 Census
Self-reported limiting long-term heath problem or disability	Not limited, daily activity limited a lot, daily activity limited a little	2011 Census
Pre-existing conditions	Dummies for cancer, cardiovascular diseases, digestive disorders, mental health conditions, metabolic disorders, musculoskeletal disorders, neurological disorders, renal disorders, respiratory disorders	HES (2017–2019)
Number of hospital admissions	0, 1, 2–3, 4–5, 6–9, 10+	HES (2017–2019)
Total days spent in hospital	0, 1, 2–4, 5–9, 10–19, 20–39, 40–69, 70+	HES (2017–2019)

a Household deprivation is defined according to four dimensions: employment (at least one household member is unemployed or long-term sick, excluding full-time students); education (no household members have at least Level 2 education, and no one aged 16–18 years is a full-time student); health and disability (at least one household member reported their health as being ‘bad’/‘very bad’ or has a long-term health problem); and housing (the household's accommodation is overcrowded, with an occupancy rating −1 or less, or is in a shared dwelling, or has no central heating). For people aged over 75 years at time of the 2011 Census, approximated social grade was imputed based on household tenure. Key worker type is defined based on the occupation and industry code. ‘Exposure to disease’ and ‘proximity to others’ are derived from the O*NET database, which collects a range of information about individuals’ working conditions and day-to-day tasks of their job. To calculate the proximity and exposure measures, the questions asked were as follows: (i) How physically close to other people are you when you perform your current job? (ii) How often does your current job require that you be exposed to diseases or infection? Scores ranging from 0 (no exposure) to 100 (maximum exposure) were calculated based on these questions using methods previously described by the ONS.

### Outcome and exposure

Deaths involving COVID-19 included those with an underlying cause, or any mention, of International Statistical Classification of Diseases and Related Health Problems 10th Revision (ICD-10) codes U07.1 (COVID-19, virus identified) or U07.2 (COVID-19, virus not identified). We analysed deaths that occurred between 2 March 2020 and 30 November 2020, which corresponds to the deaths that occurred during the first and second COVID-19 waves.

Household composition in 2020 was derived based the household composition at the time of the Census. We excluded people who died between 27 March 2011 and 1 March 2020 or had moved to a care home by 2019. To mitigate measurement error, we removed people aged 10 to 24 at the time of the Census because they were more likely to have moved out in 2020. We defined a multi-generational household to be a household in which someone aged 65 years or over on 2 March in 2020 co-resided with at least one other adult aged more than 20 years younger or with at least one child. Our household composition variable classified households in five categories: Single; Two elderly adults; Multi-generational household without dependent children; Multi-generational household with dependent children; three or more elderly adults. As sensitivity analyses, we removed people aged 10 to 19 instead of 10 to 24. We also defined a multi-generational household to be one in which someone aged 65 years or over in 2020 co-resided with at least one other adult aged more than 15 years (instead of 20 years) younger. We also used longitudinal data from the English Longitudinal Study of Ageing to estimate change in household composition among adults aged 65 or over between 2008–2009 and 2016–2017 (see Appendix for more details). We also compare estimates of the proportion of elderly adults living in multi-generational households by ethnic group in our linked data to estimates based on the 2019 Annual Population Survey.

In the mediation analysis, the exposure was self-reported ethnic affiliation based on a nine-group classification (Table 4 in Appendix 1). The two mediators were binary variables for living in a multi-generational household with or without children.

### Covariates

**Figure 1.**
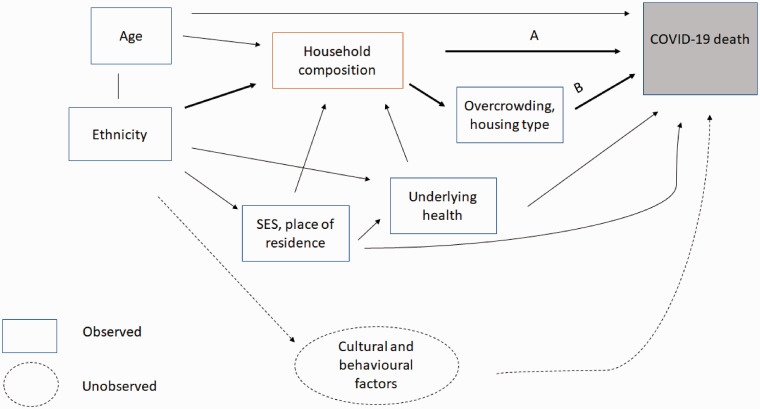
Directed Acyclic Graphs summarising the relationship between ethnicity, household composition and COVID-19 mortality. Note: When analysing whether household composition directly affects the risk of COVID-19 death, our effect of interest is A. In the mediation analysis, where we estimate the proportion of the ethnic disparity in COVID-19 that is explained by living in a multi-generational household, the effects of interest are A + B.

Demographic factors, geographical variables, socio-economic characteristics and measures of pre-pandemic health are listed in Table 4 in Appendix 1. These covariates were generally considered to be confounders of the relationship between household composition and COVID-19 mortality risk, and mediators of the ethnicity–mortality relationship (Figure 1).

### Statistical analyses

We calculated age-standardised mortality rates (ASMRs) stratified by household composition and sex, separately for COVID-19-related deaths and other deaths. The ASMRs were standardised to the 2013 European Standard Population and can be interpreted as deaths per 100,000 of the population at risk during the analysis period.

We estimated Cox proportional hazard models to assess whether the risk of COVID-19-related death varies by household type (using living with one other older adult with as the reference category) after adjusting for the geographical factors, socio-economic characteristics and measures of health listed in Table 4 in Appendix 1. These factors could confound the relationship between household composition and COVID-19-related mortality, as shown in Figure 1. We estimated separate models for men and women, as the risk of death involving COVID-19 differs markedly by sex.^7^ When fitting the Cox models, we included all individuals who died during the analysis period and a weighted random sample of those who did not (5% of White people, and 20% among ethnic minority groups), and applied case weights to reflect the original population totals. Standard errors were clustered at the household level. We assessed the proportional hazard assumption by testing for the independence between the scaled Schoenfeld residuals and time-at-risk.

We conducted a causal mediation analysis^14^ to estimate the proportion of excess risk in ethnic minority groups which is attributable to living in a multigenerational household. As a measure of inequality in COVID-19 mortality, ethnicity-specific odds ratios for COVID-19 mortality were estimated using logistic regression models, fitted to men and women separately and adjusting solely for age in the baseline model. The proportion of the difference in COVID-19 mortality rates between ethnic groups mediated by living in a multi-generational household was then estimated as the Average Causal Mediated Effect as a proportion of the age-adjusted difference in the probability of COVID-19 mortality, using a non-parametric approach^15^ (see Appendix 1 for more details). The mediator models and the full outcome model were adjusted for geographical factors (region, population density, urban/rural classification), socio-economic characteristics (IMD decile, educational attainment, social grade, household tenancy) and health (self-reported health and disability from the Census, pre-existing conditions based on hospital contacts), We did not adjust for overcrowding or housing type, as these are likely to be consequences of living in a multi-generational household rather than confounding factors (Figure 1) In addition, among elderly adults, household size and overcrowding are strongly linked to multi-generational households, because most households with more than two people are multi-generational. Confidence intervals were obtained via bootstrapping, clustered at the household level, using 500 replications. All statistical analyses were performed using R version 3.5.

## Results

### Characteristics of the study population

Characteristics of the study population are reported in Table 1. In our study population of 10,078,568 individuals in England aged 65 years or over who were not in a care home in 2019 and were still alive on 2 March 2020, just over half (54.0%) were female, the mean age was 75.2 years, and 93.9% reported being from a White ethnic background (Table 2). Over the outcome period (2 March 2020 to 30 November 2020), 39,419 (0.39%) died of COVID-19, and 227,041 (2.3%) died of other causes.

Compared with elderly adults living with one other older adult (*n* = 5,538,963), people living by themselves (*n* = 3,287,395) had a higher mean age, were more likely to be female and tended to be more deprived. Older people living in a multi-generational household without dependent children (*n* = 987,306) and with dependent children (n = 199,112) were on average younger and were more likely to be from an ethnic minority group, live in London and large urban conurbations, and tended to be more deprived than older people living with another older adult. Summary statistics stratified by ethnic groups are reported in Table 5 in Appendix 1.

**Table 5.** Distributions of study variables, stratified by household composition.

		White	Bangladeshi	Black African	Black Caribbean	Chinese	Indian	Mixed	Other	Pakistani
Mortality	COVID-19 death	0.0037	0.0098	0.0060	0.0086	0.0034	0.0057	0.0049	0.0049	0.0098
	Other death	0.0229	0.0212	0.0108	0.0234	0.0110	0.0156	0.0186	0.0127	0.0183
Household composition	Two older adults	0.5620	0.2575	0.3315	0.3810	0.4625	0.3678	0.4363	0.4110	0.2766
	Single	0.3316	0.1470	0.3325	0.4285	0.2435	0.1583	0.4011	0.2289	0.1294
	MGH without young children	0.0872	0.2705	0.1995	0.1398	0.2341	0.3446	0.1121	0.2427	0.3161
	MGH with young children	0.0131	0.3205	0.1204	0.0411	0.0490	0.1154	0.0326	0.1030	0.2721
	Other 3+ adults	0.0062	0.0045	0.0161	0.0097	0.0109	0.0139	0.0179	0.0143	0.0059
Age	Age in 2020	75.3	74.6	73.1	76.6	73.3	74.0	74.3	73.5	74.1
Sex	Female	0.5394	0.5768	0.5604	0.5831	0.5634	0.5258	0.5510	0.5377	0.5051
	Population density	3242.1	11801.4	10673.5	9425.8	6414.4	6954.4	6360.2	8039.3	8089.9
Region	East	0.1231	0.0701	0.0552	0.0562	0.1010	0.0586	0.0918	0.0712	0.0550
	East Midlands	0.0919	0.0253	0.0234	0.0537	0.0456	0.1332	0.0558	0.0395	0.0413
	London	0.0788	0.5694	0.7195	0.5680	0.3800	0.4369	0.3460	0.5476	0.2369
	North East	0.0545	0.0181	0.0046	0.0013	0.0272	0.0086	0.0269	0.0099	0.0193
	North West	0.1375	0.0935	0.0490	0.0398	0.1244	0.0584	0.1014	0.0483	0.1599
	South East	0.1772	0.0559	0.0685	0.0601	0.1536	0.0954	0.1640	0.1331	0.0959
	South West	0.1285	0.0136	0.0169	0.0266	0.0550	0.0158	0.0782	0.0319	0.0093
	West Midlands	0.1058	0.1114	0.0352	0.1526	0.0640	0.1552	0.0761	0.0817	0.2009
	Yorkshire and the Humbers	0.1027	0.0427	0.0277	0.0419	0.0492	0.0377	0.0598	0.0368	0.1814
Household deprivation	Not deprived	0.3582	0.1133	0.2998	0.2494	0.3771	0.2796	0.2939	0.3055	0.1401
	Deprived along 1 dimension	0.3855	0.3107	0.3617	0.3875	0.3727	0.3690	0.3668	0.3515	0.3276
	Deprived along 2 dimensions	0.2118	0.3685	0.2397	0.2894	0.2024	0.2738	0.2493	0.2448	0.3663
	Deprived along 3 dimensions	0.0418	0.1859	0.0868	0.0686	0.0447	0.0722	0.0806	0.0878	0.1515
	Deprived along 4 dimensions	0.0027	0.0216	0.0119	0.0051	0.0032	0.0054	0.0093	0.0104	0.0144
Urban/rural classification	Rural hamlets and isolated dwellings	0.0459	0.0011	0.0016	0.0025	0.0113	0.0041	0.0191	0.0068	0.0016
	Rural hamlets and isolated dwellings in a sparse setting	0.0044	*	*	0.0001	0.0006	0.0000	0.0018	0.0004	0.0001
	Rural town and fringe	0.1114	0.0058	0.0067	0.0079	0.0360	0.0125	0.0496	0.0205	0.0044
	Rural town and fringe in a sparse setting	0.0053	0.0003	0.0001	0.0001	0.0011	0.0002	0.0019	0.0008	0.0001
	Rural village	0.0783	0.0018	0.0023	0.0041	0.0155	0.0069	0.0338	0.0125	0.0024
	Rural village in a sparse setting	0.0055	*	*	0.0001	0.0004	0.0002	0.0018	0.0003	0.0001
	Urban city and town	0.4480	0.1887	0.1400	0.1712	0.3301	0.3100	0.3411	0.2365	0.2612
	Urban city and town in a sparse setting	0.0023	*	0.0001	*	0.0000	0.0001	0.0019	0.0002	*
	Urban major conurbation	0.2643	0.7932	0.8322	0.7823	0.5811	0.6528	0.5239	0.7054	0.6889
	Urban minor conurbation	0.0346	0.0091	0.0169	0.0319	0.0239	0.0134	0.0252	0.0166	0.0413
Approximated social grade	AB	0.1981	0.1299	0.1850	0.0927	0.1815	0.2198	0.1842	0.2250	0.1464
	C1	0.2979	0.2037	0.2919	0.2616	0.3105	0.2811	0.2886	0.2828	0.1983
	C2	0.2038	0.0796	0.1469	0.2182	0.1283	0.1405	0.1734	0.1395	0.1426
	D	0.2655	0.3739	0.2344	0.3483	0.2883	0.2958	0.2668	0.2402	0.3794
	E	0.0346	0.2129	0.1418	0.0793	0.0915	0.0628	0.0870	0.1125	0.1333
Health	Very good	0.2255	0.0505	0.2605	0.1499	0.1827	0.1309	0.2066	0.1720	0.0752
	Good	0.4506	0.2291	0.4179	0.3557	0.4764	0.4161	0.3871	0.3985	0.2913
	Fair	0.2481	0.4039	0.2285	0.3596	0.2735	0.3202	0.2785	0.2936	0.3861
	Poor	0.0628	0.2418	0.0711	0.1034	0.0572	0.1077	0.0979	0.1071	0.1945
	Very poor	0.0130	0.0746	0.0219	0.0314	0.0102	0.0251	0.0298	0.0289	0.0528
Long-term health problem or disability	Daily activities no limited	0.1188	0.3281	0.1583	0.1849	0.0709	0.1861	0.1699	0.1728	0.3014
	Daily activities limited a little	0.1921	0.3040	0.1607	0.2088	0.1671	0.2264	0.1912	0.2026	0.2769
	Daily activities limited a lot	0.6891	0.3679	0.6810	0.6063	0.7620	0.5875	0.6389	0.6246	0.4217
Hospital-based co-morbidities	Cancer	0.1029	0.0677	0.0717	0.0927	0.0599	0.0629	0.0807	0.0699	0.0636
	Cardiovascular disease	0.2808	0.3882	0.2686	0.3378	0.1858	0.3369	0.2645	0.2854	0.3759
	Digestive disorder	0.0192	0.0363	0.0129	0.0170	0.0188	0.0212	0.0206	0.0185	0.0248
	Mental health condition	0.0207	0.0476	0.0234	0.0418	0.0129	0.0223	0.0267	0.0232	0.0285
	Metabolic disorder	0.0788	0.3168	0.1335	0.1922	0.0877	0.1941	0.1128	0.1603	0.2820
	Musculoskeletal disorder	0.0996	0.1010	0.0723	0.0951	0.0463	0.0966	0.0861	0.0788	0.1078
	Neurological disorder	0.0180	0.0119	0.0122	0.0154	0.0073	0.0150	0.0176	0.0128	0.0168
	Renal disorder	0.0668	0.1214	0.0636	0.0959	0.0354	0.0768	0.0668	0.0592	0.1022
	Respiratory disorder	0.1375	0.1964	0.0919	0.1289	0.0815	0.1359	0.1406	0.1265	0.1901
Overcrowded		0.0236	0.2989	0.3104	0.1140	0.0933	0.0994	0.1091	0.1692	0.1840
IMD Decile	1	0.0611	0.3385	0.2009	0.2103	0.0960	0.0878	0.1166	0.1093	0.3153
	2	0.0665	0.2183	0.2660	0.2242	0.1068	0.1185	0.1317	0.1386	0.1894
	3	0.0772	0.1317	0.1919	0.1752	0.1123	0.1411	0.1240	0.1346	0.1314
	4	0.0895	0.0844	0.1074	0.1257	0.0978	0.1277	0.1087	0.1178	0.0978
	5	0.1022	0.0627	0.0738	0.0899	0.0990	0.1261	0.0999	0.1049	0.0784
	6	0.1122	0.0504	0.0538	0.0626	0.0984	0.1054	0.0954	0.0986	0.0609
	7	0.1203	0.0352	0.0347	0.0369	0.0939	0.0842	0.0884	0.0802	0.0391
	8	0.1228	0.0283	0.0277	0.0292	0.0871	0.0695	0.0814	0.0732	0.0339
	9	0.1247	0.0293	0.0243	0.0274	0.0977	0.0692	0.0809	0.0731	0.0281
	10	0.1236	0.0211	0.0195	0.0185	0.1111	0.0705	0.0727	0.0698	0.0257
Level of highest qualification	No qualification	0.3734	0.6659	0.1994	0.4014	0.4227	0.3765	0.3233	0.2836	0.5657
	Level 1: 1–4 GCSE/O-Level	0.0939	0.0334	0.0672	0.0892	0.0504	0.0796	0.0950	0.0654	0.0588
	Level 2: 5+ GCSE/O levels	0.1074	0.0152	0.0956	0.0854	0.0518	0.0605	0.0950	0.0720	0.0345
	Apprenticeship	0.0616	0.0017	0.0085	0.0279	0.0075	0.0121	0.0340	0.0097	0.0067
	Level 3: 2+ A Levels or equivalent	0.0679	0.0143	0.0652	0.0531	0.0314	0.0346	0.0672	0.0468	0.0247
	Level 4+: degree or above	0.2371	0.1059	0.4405	0.2262	0.2863	0.2365	0.2782	0.3242	0.1268
	Other	0.0587	0.1635	0.1236	0.1168	0.1500	0.2002	0.1073	0.1983	0.1828
Tenure of household	Owned outright	0.6322	0.2218	0.1304	0.3879	0.5506	0.5474	0.4059	0.3677	0.4723
	Owned with a mortgage	0.1889	0.3238	0.2826	0.2530	0.2139	0.3181	0.2269	0.3028	0.3242
	Shared ownership	0.0041	0.0037	0.0118	0.0057	0.0051	0.0029	0.0066	0.0055	0.0025
	Social rented from council	0.0637	0.1809	0.2491	0.1750	0.0754	0.0325	0.1348	0.1031	0.0480
	Other social rented	0.0535	0.1608	0.1710	0.1223	0.0650	0.0305	0.1081	0.0788	0.0466
	Private rented	0.0470	0.0902	0.1374	0.0472	0.0614	0.0544	0.1013	0.1230	0.0863
	Living rent-free	0.0106	0.0189	0.0177	0.0089	0.0286	0.0141	0.0165	0.0191	0.0201

MGH: multi-generational household; GCSE: General Certificate of Secondary Education.

**Figure 2.**
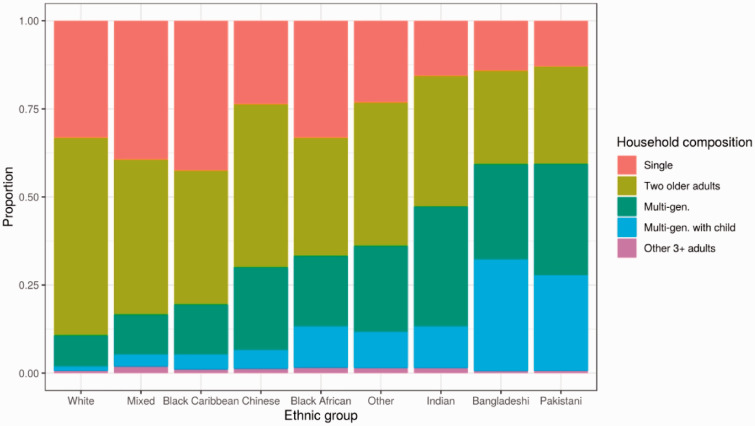
Household composition by ethnic group for people in England aged ≥ 65 years. Note: Linked 2011 Census and mortality registration data for people in England aged ≥ 65 years, excluding those living in a care home in 2019. The number of adults in the household was calculated as the number of people aged ≥ 25 years who lived in the household at the time of the Census, minus those who died between 27 March 2011 and 1 March 2020.

Figure 2 shows that household composition varied substantially between ethnic groups. Among older people, just over 10% of those of White background lived in a multi-generational household, compared to over half of Bangladeshi or Pakistani background (58.7% and 58.8%, respectively) and 45.8% of Indian background. The patterns were similar for men and women, although a larger proportion of women live by themselves (Figure 5 in Appendix 1). Similar proportions are obtained when using data from the 2019 Annual Population Survey and applying the same definition of multi-generational households (Table 6 in Appendix 1).

**Table 6.** Proportion of elderly adults (65+) living in a multi-generational household by ethnic group in 2019 Annual Population Survey.

Ethnic group	Proportion living in a multi-generational household (95% CI)
White	9.1% (8.8–9.4)
Mixed	12.3% (5.6–18.9)
Indian	36.7% (32.8–40.6)
Pakistani	55.3% (48.9–61.7)
Bangladeshi	63.9% (52.8–75)
Chinese	22.8% (13.4–32.1)
Any other Asian background	35.6% (27.6–43.7)
Black	28.3% (24.4–32.2)
Arab	16.1% (4.7–27.5)
Other ethnic group	23% (16.7–29.2)

Note: Weighted average based on 2019 Annual Population Survey; Respondents living in England.

### Household composition and death involving COVID-19

**Figure 3.**
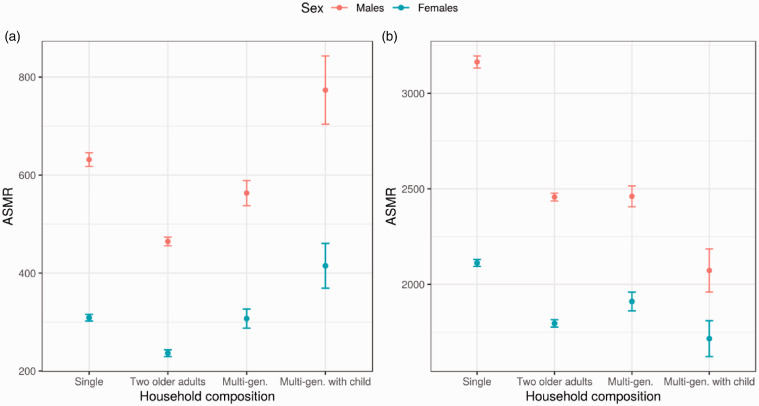
Age-standardised mortality rates per 100,000 adults aged 65 years or over, stratified by sex and household composition. Note: Deaths occurring between 2 March 2020 and 30 November 2020. 95% confidence intervals are reported. Mortality rates are standardised to the 2013 European Standard Population.

Elderly people living by themselves were more likely to have died from COVID-19 over the study period than those living with another adult. For men, the ASMRs were 632 (95% confidence interval [CI]: 618 – 646) and 465 (95% CI 456–473) per 100,000 of the population for those living by themselves and those living with another adult, respectively (Figure 3, Panel A). For women, the ASMRs were 309 (95% CI 302–316) and 236 (95% CI 230–243) per 100,000, respectively. A similar pattern is observed for deaths from other causes (Figure 3, Panel B).

There was a positive association between the risk of COVID-19 death and living a in multi-generational household. Both elderly men and women living a multi-generational household without school-age children were more likely to die from COVID-19 than elderly people living with another elderly adult (ASMR 563 [95% CI 538–589] per 100,000 for men, 307 [95% CI 288–327] for women), with the risk of death being greater still if there were children in the household (ASMR 773 [95% CI 704–843] per 100,000 for men, 415 [95% CI 369–461] per 100,000 for women). The risk of COVID-19 mortality was higher in men than that in women across all the household compositions (Figure 3). There was no clear relationship between living in a multi-generational household and mortality from other causes.

Adjusting for individual- and household-level characteristics (including age, geographical factors, socioeconomic characteristics and measures of pre-pandemic health) reduced the estimated differences in COVID-19 mortality rates between elderly adults living in different types households (Table 2). Adjusting for socioeconomic factors, such as IMD decile, household deprivation, educational attainment, social grade and household tenancy, had the strongest effect of the hazard ratio (Table 7 in Appendix 1). However, even after adjusting for these characteristics, living in a multi-generational household, especially with children, remained associated with an increased risk of COVID-19-related death. Compared to living with another elderly adult aged 65 years or above, the rate of COVID-19-related death was 1.16 (95% CI 1.07–1.25) and 1.21 (95% CI 1.06–1.38) times greater for elderly women living in a multi-generational household without and with children, respectively. For elderly men, after adjusting for individual and household characteristics, living in a multi-generational household without children was associated with a 1.07 (95% CI 1.01–1.13) times greater risk of COVID-19-related death and living in a multi-generational household with children with a 1.17 (95% CI 1.06–1.30) times greater risk.

**Table 7.** Hazard ratios for COVID-19-related death for elderly adults (aged ≥ 65 years) in England, compared to living in a household with one other older adult, with different adjustments, stratified by sex.

	Age adjusted	+ Geographical factors	+ Socio- demographic factors	+ Pre-pandemic health	+Housing	+Occupational exposure
	A. Men					
Single	1.30 [1.24–1.37)	1.19 [1.14–1.25)	1.07 [1.02–1.12)	1.06 [1.01–1.12)	1.07 [1.01–1.12)	1.07 [1.01–1.13)
Multi-gen. without children	1.77 (1.62–1.94)	1.49 (1.36–1.63)	1.09 (0.99–1.20)	1.15 (1.05–1.28)	1.17 (1.06–1.30)	1.18 (1.06–1.30)
Multi-gen. with children	0.89 (0.75–1.07)	0.83 (0.69–1.00)	0.70 (0.58–0.84)	0.98 (0.81–1.18)	1.00 (0.82–1.20)	1.00 (0.83–1.20)
3+ older adults	1.37 (1.33–1.41)	1.28 (1.24–1.32)	1.15 (1.12–1.19)	1.14 (1.10–1.18)	1.13 (1.09–1.17)	1.12 (1.08–1.17)
Concordance	0.740	0.747	0.764	0.826	0.826	0.826
	B. Women					
Single	1.32 (1.23–1.42)	1.23 (1.15–1.32)	1.12 (1.04–1.20)	1.15 (1.07–1.24)	1.16 (1.07–1.25)	1.16 (1.07–1.25)
Multi-gen. without children	1.86 (1.66–2.08)	1.57 (1.40–1.77)	1.18 (1.04–1.33)	1.19 (1.04–1.35)	1.21 (1.06–1.38)	1.21 (1.06–1.38)
Multi-gen. with children	0.72 (0.57–0.92)	0.69 (0.54–0.88)	0.59 (0.47–0.75)	0.83 (0.65–1.07)	0.85 (0.66–1.09)	0.85 (0.66–1.09)
3+ older adults	1.29 (1.24–1.34)	1.25 (1.20–1.30)	1.13 (1.08–1.17)	1.11 (1.06–1.16)	1.10 (1.06–1.15)	1.10 (1.05–1.15)
Concordance	0.755	0.763	0.782	0.851	0.852	0.852

Hazard ratios compared to living in a household with one other older adult. Geographical factors: region, population density, urban/rural classification. Socioeconomic characteristics: ethnicity, IMD decile, household deprivation, educational attainment, social grade, household tenancy; pre-pandemic health: self-reported health and disability from the Census, pre-existing conditions based on hospital contacts, number of hospital admissions, total days spent in hospital; overcrowding; Housing: overcrowding and property type. Occupational exposure: Maximum of ‘exposure to disease’ score within each household; Maximum of ‘proximity to others’ score within each household.

Using the ASMRs for women living with another elderly adult as the baseline risk, these hazard ratios imply that living in a multi-generational household without children would increase the ASMRs from 236 to 274 death per 100,000 people, and living in a multi-generational household with children to 286 death per 100,000 people. For men, living in a multigenerational household without children is expected to increase the ASMRs from 465 to 498 death per 100,000 people, and living in a multi-generational household with children to 544 death per 100,000 people.

The rate of COVID-19-related death was also 1.13 (95% CI 1.09–1.17) times greater for elderly men living alone than for those living with another older adult, and 1.10 (95% CI 1.06–1.15) times greater for elderly women. The results were similar in the sensitivity analyses using different definitions of household composition (Table 8 in Appendix 1).

**Table 8.** Hazard ratios for COVID-19-related death for elderly adults (aged ≥65 years) in England, compared to living in a household with one other older adult, using different definitions of household composition, stratified by sex.

	Men		Women	
	Def. A1	Def. A2	Def. A1	Def. A2
Single	1.13	1.13	1.11	1.1
	(1.10–1.17)	(1.09–1.17)	(1.06–1.15)	(1.06–1.15)
Multi-generational household without children	1.07	1.04	1.16	1.16
	(1.02–1.12)	(0.99–1.10)	(1.08–1.24)	(1.08–1.24)
Multi-generational household with children	1.17	1.17	1.21	1.21
	(1.07–1.29)	(1.06–1.29)	(1.07–1.37)	(1.06–1.38)
3+ elderly adults	0.98	1.02	0.83	0.76
	(0.82–1.18)	(0.84–1.23)	(0.66–1.06)	(0.58–0.99)
Observations	405,182		443,650	
Concordance	0.826	0.826	0.852	0.851

Note: Def A1: we derived household composition in 2020 based on the number of adults aged 20 years (instead of 25 years); Def A2: multi-generational household as household with someone aged 65 years or over in 2020 co-resided with at least one other adult aged more than 15 years (instead of 20 years) younger. Hazard ratios compared to living in a household with one other older adult. Fully adjusted Cox regression models include geographical factors (region, population density, urban/rural classification), ethnicity, socioeconomic characteristics (IMD decile, household deprivation, educational attainment, social grade, household tenancy), health (self-reported health and disability from the Census, pre-existing conditions based on hospital contacts, number of hospital admissions, total days spent in hospital), a measure for overcrowding and property type.

We tested the proportional hazard assumption by testing for the independence between the scaled Schoenfeld residuals and time-at-risk. For men, the test failed to reject the independence for the exposure (p = 0.112 for men), suggesting that the proportional hazard assumption was unlikely to be violated. However, for women, the test suggested that the proportional hazard assumption was likely to be violated (p = 0.002), However, as shown by the smoothed Schoenfeld residuals for each group (Figure 6 in Appendix 1), the deviation from the estimated log-hazard ratio is small, and the 95% confidence intervals around the smoothed Schoenfeld residuals always included the estimated log-hazard ratio, suggesting that violation of the proportional hazard assumption was unlikely to substantially affect our main results.

**Figure 4.**
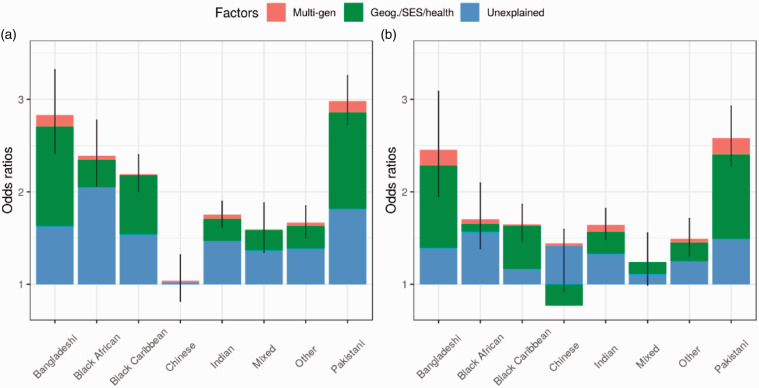
Decomposition of odds ratios for COVID-19 mortality among elderly adults (aged ≥ 65 years) across ethnic groups, stratified by sex. Note: The overall height of the bar corresponds to the odds ratio (OR), relative to the White population, based on a logistic regression model adjusted for age. Error bars are 95% confidence intervals. The proportion of the age-adjusted ORs explained by living in a multi-generational household were calculated through a mediation analysis. The unexplained part corresponds to the ORs from a model adjusted for age, geographical factors (region, population density, urban/rural classification), socioeconomic characteristics (IMD decile, household deprivation, educational attainment, social grade, household tenancy), and health (self-reported health and disability from the Census, pre-existing conditions based on hospital contacts, number of hospital admissions, total days spent in hospital).

**Figure 5.**
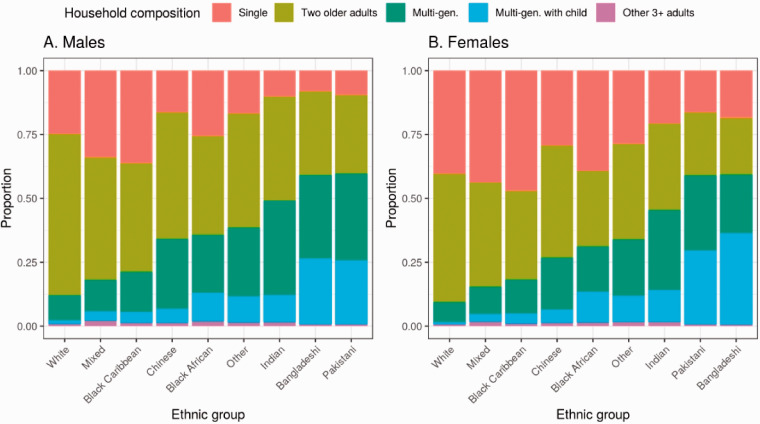
Household composition by ethnic group for people in England aged ≥ 65 years, stratified by sex. Note: Linked 2011 Census and mortality registration data for people in England aged ≥ 65 years, excluding those living in a care home in 2019. The number of adults in the household was calculated as the number of people aged ≥ 16 years who lived in the household at the time of the Census, minus those who died between 27 March 2011 and 1 March 2020.

**Figure 6.**
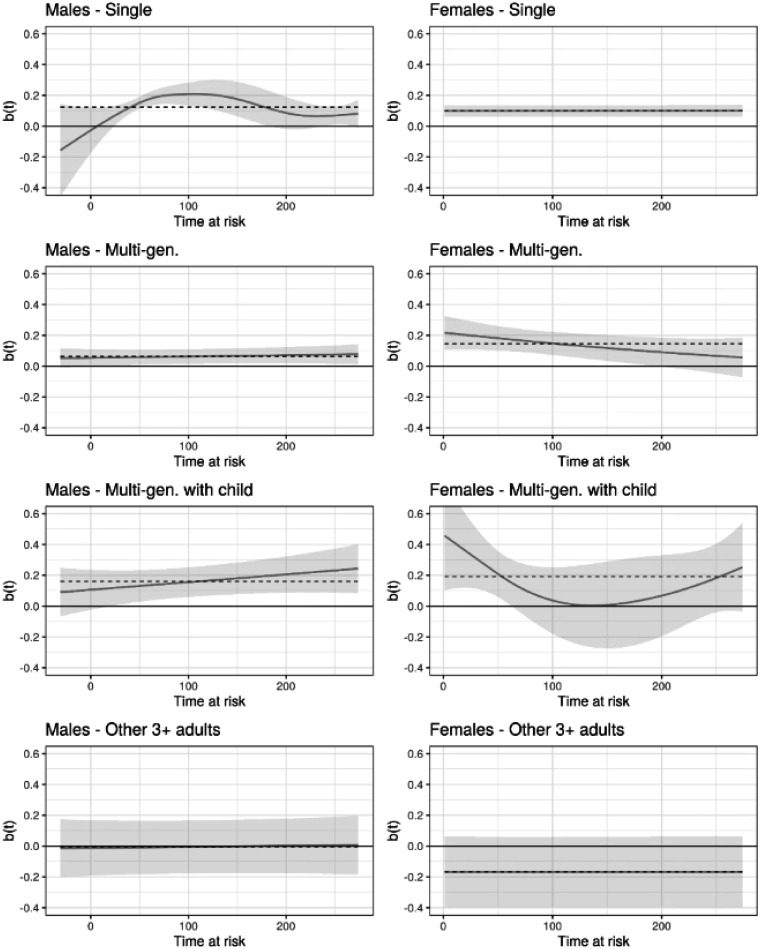
Smoothed Schoenfeld residuals for household composition from the fully adjusted model, stratified by sex. Note: Fully adjusted Cox regression models include geographical factors (region, population density, urban/rural classification), ethnicity, socioeconomic characteristics (IMD decile, household deprivation, educational attainment, social grade, household tenancy), health (self-reported health and disability from the Census, pre-existing conditions based on hospital contacts, number of hospital admissions, total days spent in hospital), a measure for overcrowding and property type. Dotted line shows the log-hazard ratio from the model. Residuals are smoothed with a generalised additive model. Confidence intervals are at the 95% level.

### Living in a multi-generational household as a mediator for the disparity in COVID-19 death between ethnic groups

Figure 4 shows the decomposition of the age-adjusted odds ratios of COVID-19 death for ethnic minority groups compared to those of white ethnic group into and Three parts: (i) the part explained by living in a multi-generational household (in red); (ii) the part explained by other individual and household characteristics, such as geographical factors, socioeconomic factors and pre-pandemic health (in green); and (iii) a residual component that is not explained by our model (in blue).

Among people aged 65 years or over, those from all ethnic minority groups except Chinese were at greater risk of COVID-19-related death than those from the White population. Compared to men and women from White ethnic group, the odds of COVID-19 death were 2.98 (95% CI 2.73–3.26) and 2.58 (95% CI 2.28–2.93) times greater for men and women from Pakistani ethnic background. The odds of death were also notably greater for people of Bangladeshi, Black African, Black Caribbean or Indian ethnic background than the White population, with odds ratios of 2.83 (95% CI 2.42–3.32), 2.39 (95% CI 2.06–2.77), 2.19 (95% CI 2.00–2.40) and 1.75 (95% CI 1.26–1.90), respectively, for men and 2.45 (95% CI1.95–3.09), 1.71 (95% CI 1.39–2.09), 1.65 (95% CI 1.46–1.86) and 1.64 (95% CI 1.48–1.86), respectively, for women.

Living in a multi-generational household did not explain much of the difference in COVID-19 mortality rates among elderly men. It accounted for 6.9% (95% CI 2.7–11.3) of the excess risk of COVID-19 mortality for men of Pakistani background, 6.0% (95% CI 2.3–9.9) for men of Indian background and 6.3% (95% CI 2.6–10.2) for men of Pakistani background. It did explain a larger proportion the difference in risk between elderly women of South Asian background and White elderly women Living in a multi-generational household accounted for 11.6% (95% CI 5.9–18.0) of the excess risk of COVID-19 mortality for women of Indian background, and 11.5% (95% CI 4.0–20.0) and 11.1% (95% CI 4.8–17.5) for women of Bangladeshi and Pakistani background (see Table 9 in Appendix 1 for full results). The results were similar in the sensitivity analyses using different definitions of household composition (Table 10 in Appendix 1).

**Table 9.** Proportion of difference in COVID-19 mortality rates between ethnic groups mediated by living in a multi-generational household, stratified by sex.

	Multi-gen with young children		Multi-gen without young children		Multi-gen (total)	
	Prop.	95% CI	Prop.	95% CI	Prop.	95% CI
Women						
Bangladeshi	0.089	(0.014–0.167)	0.027	(0.013–0.044)	0.115	(0.040–0.200)
Black African	0.044	(0.007–0.097)	0.026	(0.012–0.054)	0.070	(0.029–0.138)
Black Caribbean	0.010	(0.002–0.020)	0.011	(0.005–0.020)	0.022	(0.010–0.036)
Chinese	0.033	(−0.318–0.184)	0.085	(−0.892–0.551)	0.117	(−1.142–0.753)
Indian	0.046	(0.007–0.086)	0.070	(0.033–0.115)	0.116	(0.059–0.180)
Mixed	0.013	(−0.071–0.122)	0.008	(−0.036–0.077)	0.020	(−0.104–0.202)
Other	0.043	(0.007–0.091)	0.044	(0.020–0.078)	0.087	(0.042–0.153)
Pakistani	0.071	(0.011–0.128)	0.039	(0.018–0.062)	0.111	(0.048–0.175)
Men						
Bangladeshi	0.051	(0.013–0.092)	0.018	(0.001–0.034)	0.069	(0.027–0.113)
Black African	0.020	(0.005–0.037)	0.010	(0.001–0.019)	0.030	(0.013–0.051)
Black Caribbean	0.006	(0.001–0.011)	0.004	(0.000–0.009)	0.010	(0.004–0.017)
Chinese	0.122	(−0.390–0.446)	0.231	(−1.034–0.942)	0.353	(−1.550–1.395)
Indian	0.027	(0.007–0.049)	0.033	(0.003–0.063)	0.060	(0.023–0.099)
Mixed	0.006	(0.001–0.015)	0.002	(0.000–0.007)	0.009	(0.003–0.019)
Other	0.028	(0.006–0.051)	0.021	(0.002–0.043)	0.049	(0.020–0.082)
Pakistani	0.045	(0.011–0.079)	0.018	(0.001–0.035)	0.063	(0.026–0.102)

Note: Proportion of difference in COVID-19 mortality between ethnic group mediated by living in a multi-generational household is estimated as the Average Causal Mediated Effect (ACME) as a proportion of the age-adjusted difference in the probability of COVID-19 death. The ACME is derived based on models that adjust for geographical factors (region, population density, urban/rural classification), socioeconomic characteristics (IMD decile, household deprivation, educational attainment, social grade, household tenancy), and health: (self-reported health and disability from the Census, pre-existing conditions based on hospital contacts). 95% confidence intervals are obtained via bootstrapping, using 500 replications.

**Table 10.** Proportion of difference in COVID-19 mortality rates between ethnic groups mediated by living in a multi-generational household using different definitions of household composition, stratified by sex.

	Men				Women	
	Main def	Def. A1	Def. A2	Main def	Def. A1	Def. A2
Bangladeshi	4.5%	6.9%	6.6%	11.5%	11.5%	13.2%
Black African	1.6%	3.1%	3.0%	7.0%	7.0%	7.8%
Black Caribbean	0.5%	1.0%	0.9%	2.2%	2.0%	2.2%
Chinese	4.6%	35.4%	29.2%	11.7%	11.7%	12.4%
Indian	3.3%	6.0%	4.8%	11.6%	11.5%	11.9%
Mixed	0.4%	0.9%	0.8%	2.0%	2.0%	2.3%
Other	2.3%	4.9%	4.4%	8.7%	8.7%	9.7%
Pakistani	5.1%	6.3%	5.8%	11.1%	11.0%	12.0%

Note: Main definition of multi-generational household: someone aged 65 years or over on 2 March in 2020 co-resided with at least one other adult aged more than 20 years younger (and at least 25 in 2011), or with at least one child. Def A1: we derived household composition in 2020 based on the number of adults aged 20 years (instead of 25 years); Def A2: multi-generational household as household with someone aged 65 years or over in 2020 co-resided with at least one other adult aged more than 15 years (instead of 20 years) younger. Proportion of difference in COVID-19 mortality between ethnic group mediated by living in a multi-generational household is estimated as the Average Causal Mediated Effect (ACME) as a proportion of the age-adjusted difference in the probability of COVID-19 death. The ACME is derived based on models that adjust for geographical factors (region, population density, urban/rural classification), socioeconomic characteristics (IMD decile, household deprivation, educational attainment, social grade, household tenancy) and health (self-reported health and disability from the Census, pre-existing conditions based on hospital contacts).

## Discussion

### Principal findings

This paper makes two contributions to the research on COVID-19. First, we find that among elderly adults, household composition is associated with COVID-19 mortality, even after adjusting for a range of sociodemographic factors and measures of health. Our results indicate that compared to those living in a two older adult household, elderly adults, especially women, living in a multi-generational household are at greater risk of COVID-19 death. Living alone is also associated with elevated COVID-19 mortality. Second, we find that living in a multi-generational household explains around 11% of the excess COVID-19 mortality risk for women of South Asian background, but little for men or people from other ethnic groups.

### Comparison with related studies

Our results are consistent with emerging evidence that household size is associated with the risk of infection,^16,17^ and that elderly adults tend to be at greater risk of household transmission.^18,19^ Older people living in large household tend to live in multi-generational households, co-habiting with younger adults and children. There is some evidence that, among elderly adults, living with dependent children is not strongly associated with the risk of COVID-19 infection or adverse outcomes.^20^ While our results indicate that elderly adults living in a multi-generational household are at greater risk of COVID-19 death compared to those living with another older adult, we find little difference in risk between older people living in a multi-generational household with or without young children. Our results are consistent with a recent study using Swedish data, which show that for elderly adults, living with a working-age adult was associated with increased COVID-19 mortality.^21^

Several studies have analysed ethnic differences in COVID-19 infection and mortality.^3,4,6–8^ Although we focus on elderly adults only, we find that almost all ethnic minority groups were at higher risk of COVID-19 deaths compared to the White population, and that the differences were attenuated once we adjusted for a range of geographical factors, sociodemographic characteristics and co-morbidities. We improve the existing evidence on ethnic inequalities in COVID-19 mortality by using a causal mediation approach to quantify the importance of living in a multi-generational household.

### Mechanisms

Our results suggest that older people are placed at increased exposure to infection by living with younger adults rather than young children. After adjusting for confounding factors, we find that the risk of COVID-19 death is similar among elderly adults living in a household with young children and those living in a household with younger adults only. This could be due to schools having been closed for a substantial proportion of the period at risk. The increased risk is likely to be driven by co-residing with younger adults, who have a higher risk of infection than older people.^17^ Younger adults are likely to be at increased risk of exposure because of work, as evidence suggests that in England people who are working were at greater risk of infection compared to people not in employment, especially if they were working in patient or client-facing occupations.^17,22,23^ The interaction between job characteristics and household composition is likely to account for some of the elevated mortality among ethnic minority groups in the USA.^24^

Elderly adults living by themselves were also found to be at greater risk of COVID-19 death than those living with another older adult. During the COVID-19 pandemic, older people living alone were more likely to have received help from carers, including informal helpers, than people living with another older adult.^25^ These frequent contacts with people from different households could increase the risk of being exposed to the virus.

We find that living in a multi-generational household explains around 11% of the excess COVID-19 mortality risk for women of South Asian background, but little for men, despite a similar proportion of them living in a multi-generational household. Women spend more time at home than men and still do the majority of unpaid housework,^26^ which could increase the risk of household transmission. However, further research would be needed to understand the mechanisms driving our results.

### Strengths and limitations

The primary strength of our study lies in the use of a unique linked population-level dataset which combine the 2011 Census with death registration data and hospital records. Unlike data based solely on health records, our study dataset contains a broad range of information on demographic, socio-economic, and household characteristics, including occupation. Unlike sample survey data, it contains millions of observations covering the entire population of interest, allowing us to examine both the association between household composition and COVID-19 mortality and also whether living in a multi-generational household explains some of the disparity in COVID-19 mortality between ethnic groups. We were able to examine differences between disaggregated ethnic minority groupings rather than high-level categories of South Asian, Black and Other.

The main limitation of our study is that household composition is likely to be imprecisely measured. While household composition is based on a detailed and accurate measurement taken in 2011, we could only identify changes since then due to death of household members or a move to a care home. While we took several steps to limit the measurement error, such as focusing on elderly adults, including only adults aged 25 or over and children aged 0 to 9 at the time of the census in our definition of household composition, our household composition measure may not reflect current living circumstances of everybody in our population of interest. To mitigate concerns about measurement error, we showed that our results are robust to using different definitions of household composition. Nonetheless, measurement error is likely to attenuate the explanatory power of household composition in our models. In addition, while we have used a causal mediation approach, our analysis remains based on observational data and therefore residual confounding is likely. Another limitation is that our statistical approach assumes that the effect of living in different types of household composition is the same across ethnic groups.

## Conclusions

Elderly adults living in multi-generational households are at elevated risk of experiencing harms from COVID-19 compared to elderly adults living with people of the same age. However, there has been little focus on implementing effective interventions (such as creating plans to effectively isolate and improving ventilation within the home) to reduce transmission risk within the household.^27^ Relevant public health interventions should be directed at communities where multi-generational households are highly prevalent. Living in a multi-generational household explains some of the excess COVID-19 mortality risk for women of South Asian background, but little for men or people from other ethnic groups. Further research is needed to explain the difference in COVID-19 mortality between ethnic groups.

## Declarations

**Ethics approval:** Ethical approval was obtained from the National Statistician’s Data Ethics Advisory Committee (NSDEC(20)12).

**Guarantor:** VN.

**Contributorship:** VN, NI, DA, CL and KK contributed to the study conceptualisation and design. VN lead the preparation of the study data and performed the statistical analyses. AS assisted with the preparation of the data. All authors contributed to interpretation of the results. VN drafted the manuscript. DA, NI, CG, VK, RM, MA, PG, AJ, AB, SG and KK contributed to the critical revision of the manuscript. All authors approved the final manuscript. The corresponding author attests that all listed authors meet authorship criteria and that no others meeting the criteria have been omitted.

**Data availability statement:** Under the provisions of the Statistics and Registration Service Act 2007, the linked 2011 Census data used in this study are not permitted to be shared.

## Appendix 1

### Change in household composition in the English Longitudinal Study of Ageing

Using a measure of household composition based on outdated information from the 2011 Census may introduce some measurement error. To quantify this measurement error, we used data from wave 4 (2008–2009) and wave 8 (2016–2017) of the English Longitudinal Study of Ageing (ELSA). We used core members of the study who were interviewed in wave 4 and wave 8 of ELSA, were aged 65 years or older in the year of their wave 4 interview and lived in a private household. We identified those who lived with their adult children (aged 25 or over in wave 4) in wave 4 and in wave 8 and estimated the proportion who had a different co-residence pattern in wave 4 and 8. The mean period between wave 4 and wave 8 interview was 7.98 years (SD = 0.07 years).

Most people (93.1%) had the same co-residence pattern in 2008–2009 and 2016–2017. Nearly all (96.6%) of the people who did not live with an adult child in wave 4 did not live with an adult child in wave 8. The majority (61.2%) of people who lived with an adult child in wave 4 also lived with an adult child in wave 8.

### Estimating the Average Causal Mediated Effect

The Average Causal Mediated Effect (ACME) of living in a multi-generational household for ethnic group k compared to the White group, *δ*(*k*) is estimated as:

$$\begin{array}{c} \bar{\delta (k)}=\text{E}\{[\mathrm{Pr}(\mathrm{MGH}=1|\mathrm{Ethnic}=k,X)\\ -\mathrm{Pr}(\mathrm{MGH}=1|\mathrm{Ethnic}=\mathrm{White},X)]\\ {}[\mathrm{Pr}(\mathrm{Cdeath}=1|\mathrm{MGH}=0,\mathrm{Ethnic}=k,X)\\ -\mathrm{Pr}(\mathrm{Cdeath}=1|\mathrm{MGH}=1,\mathrm{Ethnic}=k,X)\}] \end{array}$$

where *MGH* indicates if individual *i* lives in a multi-generational household, *Ethnic* is the ethnic group and *X* is a vector of factors likely to confound the relationship between the mediator and the outcome. *X* includes geographical factors (region, population density, urban/rural classification), socioeconomic characteristics (IMD decile, household deprivation, educational attainment, social grade, household tenancy) and health (self-reported health and disability from the Census, pre-existing conditions based on hospital contacts, number of hospital admissions, total days spent in hospital).

To estimate each component of $\bar{\delta (k)}$, we use predicted probabilities based on logistic regression models.

The total estimated difference in probability of COVID-19 death between ethnic group k and the White ethnic group is given by

$$\begin{array}{c} \bar{\tau (k)}=E\{\mathrm{Pr}(\mathrm{Cdeath}=1|\mathrm{Ethnic}=k,\mathrm{age})-\mathrm{Pr}(\mathrm{Cdeath}=1|\mathrm{Ethnic}=\mathrm{White},\mathrm{age})\} \end{array}$$

The proportion of the difference in the probability to die from COVID-19 between ethnic groups that is mediated by living in a multi-generational household is given by the ACME, $\bar{\delta (k)}$, as a proportion of the total effect $\bar{\tau (k)}$. We then used this proportion to decompose the age-adjusted odds ratios.
